# A Prospective Observational Study on Clinical Profile and Visual Outcomes in Neovascular Age-Related Macular Degeneration

**DOI:** 10.7759/cureus.52731

**Published:** 2024-01-22

**Authors:** Ramamani Dalai, Snigdha S Bedant, Rajashree Rout, Bijnya B Panda

**Affiliations:** 1 Ophthalmology, Fakir Mohan Medical College and Hospital, Balasore, IND; 2 Ophthalmology, Srirama Chandra Bhanja Medical College and Hospital, Cuttack, IND; 3 Ophthalmology, Saheed Laxman Nayak Medical College and Hospital, Koraput, IND; 4 Ophthalmology, All India Institute of Medical Sciences, Bhubaneswar, IND

**Keywords:** intravitreal injections, retinal pigment epithelium, optical coherence tomography, choroidal neovascularization, age-related macular degeneration

## Abstract

Background and objectives

Over the years, several treatment options have been developed for neovascular age-related macular degeneration (AMD), the most notable being intravitreal injections of anti-vascular endothelial growth factor drugs. The rationale for treating neovascular AMD is to preserve and improve central vision, enhance the quality of life for affected individuals, stabilize or improve vision, and prevent further structural damage to the macula. The objective of the present study was to evaluate the clinical course of different disease types of neovascular age-related macular degeneration and their treatment response to anti-vascular endothelial growth factor (anti-VEGF) injections.

Methods

This prospective observational study was conducted at a tertiary care referral hospital in Eastern India during October 2019 and September 2021. Patients diagnosed with neovascular AMD attending our Outpatient department and retina clinic were recruited for the study. An experienced ophthalmologist examined all patients, meeting the inclusion criteria. The clinical profile, including initial best corrected visual acuity (BCVA), ophthalmoscopic, fluorescein angiographic, and optical coherence tomography (OCT) findings of different patterns of neovascular AMD, were collected and analyzed. Patients were subjected to intravitreal Ranibizumab every month for three months and then on a when-required basis. Visual outcomes were recorded at each follow-up, and a comparison was done between initial and final visual acuity. Descriptive statistics were used for analysis, with p< 0.05 taken as statistically significant.

Results

A total of 72 patients were included in the study. Fundus fluorescein angiography revealed that 52.78% were classic, 15.28% were minimally classic, and 31.94% were of occult variety. 41.66% of lesions were subfoveal in location, 47.22% were juxtafoveal, and 11.11% lesions were extrafoveal in location. The mean BCVA was Log MAR (Logarithm of the Minimum Angle of Resolution) 1.061±0.25. The average number of intravitreal Ranibizumab injections given to each eye was five. BCVA of patients after the third injection was log MAR 0.818±0.296. There was a significant improvement in mean BCVA from baseline 1.061±0.254 to 0.787±0.317 after the study (p-valve: p<0.05). After the first injection, 49 patients (68.05%) experienced an initial improvement of at least one line, 20 patients (27.77%) did not exhibit any improvement, and 3 patients (4.16%) had a decline of one line in Snellen's visual acuity chart. Over the follow-up period,10 showed improvement in 1 line in the Snellen chart after subsequent injection. At the end of the study, six patients showed no change, and four patients showed deterioration after the completion of injections. No adverse events were noted during the study period.

Conclusions

Intravitreal Ranibizumab is effective in improving visual outcomes in treatment-naïve individuals with neovascular age-related macular degeneration. The decision for repeat intravitreal anti-VEGF injection should be based on OCT findings of subretinal fluid, pigment epithelial detachment, and cystoid macular edema as an indicator of disease activity. This can also lessen the number of intravitreal injections and morbidity in these patients.

## Introduction

Age-related macular degeneration (AMD) stands as a prominent contributor to permanent vision loss among the elderly population, comprising 8.7% of total global blindness cases [1]. It is not merely an eye condition but has wide-ranging and significant consequences for the affected individuals, their families, and society as a whole, making it a pressing public health concern [2]. Age-related macular degeneration (AMD) is classified into two main types: the neovascular or "wet" form and the non-neovascular or "dry" form, with some cases presenting a combination of both. These conditions can lead to partial or complete loss of central vision. The "wet" form, also known as neovascular AMD, is responsible for approximately 90% of the blindness that results from AMD. The single most significant risk factor for AMD is aging [2,3]. Consequently, with an aging global population and shifts toward Western dietary and lifestyle patterns, the number of individuals affected by AMD is expected to rise from 196 million in 2020 to 288 million by 2040 [4,5]. In India, the overall prevalence of AMD varies, with the lowest occurrence in the western region (1.4%) and the highest in the southern region (3.1%) [6].

For accurate diagnosis and staging of AMD, an ophthalmic examination is essential. This includes fundus imaging to detect symptoms such as drusen deposits, changes in the pigmentation of the retinal pigment epithelium (RPE), and degeneration of the neural retina or the presence of exudative changes. Fluorescein angiography, which visualizes blood vessels, helps confirm the presence or absence of choroidal neovascularization (CNV). The staging of AMD as early, intermediate, or advanced is determined by the severity of symptoms, such as the number and size of drusen, pigmentary changes, and whether choroidal neovascularization is present. A variety of risk factors influence neovascular AMD. Non-modifiable risks include older age, female sex, genetic predispositions, Caucasian ethnicity, and light iris color [ 7,8]. Modifiable risk factors encompass smoking, higher body mass index (BMI), alcohol consumption, and dietary choices. Diets such as the Mediterranean or Oriental are associated with a lower risk of advanced and nvAMD, while a Western diet correlates with a higher risk of advanced AMD [9]. The cornerstone of wet AMD treatment is anti-VEGF therapy. Newer treatments have been developed, including Brolucizumab (Beovu), Ranibizumab (Lucentis), aflibercept (Eylea), and the off-label use of bevacizumab (Avastin) [10]. Specifically, Ranibizumab (Lucentis) is a 48-kD Fab fragment derived from the A4.6.1 antibody, which is particularly effective in binding and neutralizing various isoforms of VEGF-A [11,12].

## Materials and methods

Study overview

This study was an observational, hospital-based prospective study conducted at a tertiary care eye hospital in the eastern part of India from October 2019 to September 2021.

Study population

All consecutive patients aged >/=50 years visiting the retina clinic during the study period diagnosed as having neovascular AMD and treatment-naive were included.

Exclusion criteria

Prior treatment for AMD, corneal, lenticular, or vitreous pathologies that prevent good quality angiogram or OCT, other co-existing ocular conditions affecting vision like diabetic retinopathy, glaucoma, etc.

Ethical considerations

Institutional Ethics Committee Clearance (vide IEC application number 666 dated 10.10.2019). Before screening and definitive examination, the study was explained in detail to all the participants. Verbal or written consent was obtained from the participants.

Study procedure

Experienced ophthalmologists performed a comprehensive ophthalmic evaluation, and findings were noted in a preformed proforma. The evaluation included best corrected visual acuity (BCVA), Slit lamp examination, Intraocular pressure, Fundus examination by Indirect ophthalmoscopy for a wider field of view, 90D lens, Fundus photography, Fundus fluorescein angiography (FFA), and Optical coherence tomography(OCT). The BCVA was measured using a Snellen chart at 6 meters with an illuminated cabinet. FFA was done every three months. Central retinal thickness (CRT) of 0.1 mm was assessed using optical coherence tomography (OCT). We performed OCT using Spectralis SD-OCT (Heidelberg Engineering, Heidelberg, Germany). Fast macular thickness scans were performed over the macula within a 6 mm cube scan centered on the fovea. It generated a macular retinal map from six consecutive fast diagonal scans measuring 0.6 mm, each intersecting at the fovea. The OCT was conducted every month.

Eyes were treated with monthly injections of Ranibizumab for three initial doses. After this period, patients were evaluated monthly with OCT to determine the need for repeat therapy. The clinical data was recorded. BCVA endpoints at the end of the study were: 1. Number of patients with ≤ 3-line loss; 2. Number of patients within 2-line gain; 3. A number of patients with ≥ 3-line gain.

Response to treatment on OCT examination was described as a decrease of 100μm in CRT, a partial response if there will be a decrease of less than 100μm in CRT, and no response if CRT changes by less than 50 μm.

Intravitreal injection procedure

After dilating the pupil and applying topical anesthesia under aseptic measures, Ranibizumab (Lucentis) 0.5 mg was injected slowly at an area 3.5mm to 4mm (pseudo phakic and phakic respectively) posterior to the limbus, avoiding the horizontal meridian & aiming towards the center of the globe and the needle was removed slowly. For subsequent intravitreal injections, the sclera site was rotated so that the same site was not injected repeatedly. Patients were monitored during the week following the injection. FFA: Pupil was dilated with tropicamide eye drops. Fundus photographs of the macular region were taken by a fundus camera, followed by red-free photographs of the same area. Fluorescein 20% solution (3ml ampoule) was injected rapidly into the antecubital vein, avoiding extravasations. Luminescence appears in the retina and choroidal vessels in 9-14 seconds. Photos were taken quickly to capture the initial phase of AV filling for 1 minute, focusing on the eye with suspected CNV. The more affected eye was photographed first. After capturing the initial AV filling, the focus shifted to the other eye to take images. Mid-phase images of both eyes were taken, and images for 10 minutes were captured to get late-phase images. FFA was interpreted regarding the extent of lesions, location (extrafoveal, juxta foveal, subfoveal), and angiographic pattern (classic, occult). Angiograms were also evaluated for blocked fluorescence, which did not correspond to hemorrhage or serous detachment of RPE.

Assessments

After three initial ranibizumab injections, patients were evaluated monthly with OCT to determine the need for repeat therapy. FFA was repeated for three months to look for the activity of CNV as evidenced by the fresh leak.

Sample size calculation

The sample size comprised consecutive patients during the study period who met the inclusion criteria.

Statistical analysis

Best corrected Visual acuity (BCVA) was converted to the logarithm of the minimum angle of resolution (logMAR) for statistical analysis. The primary outcome measures were BCVA and central macular thickness measured by optical coherence tomography at each follow-up after 4 weeks of intravitreal injection. Descriptive statistical analysis was performed, with continuous measurements presented as Mean ± Standard Deviation (SD), while categorical measurements were presented as frequencies and percentages. The student t-test was used to compare the means of the groups. Statistical significance was considered to be significant at a p-value of <0.05.

## Results

The study included 72 patients diagnosed with neovascular AMD. The predominant age group affected was 70-79 (41.66%), with male predominance (62.5%). Smoking history was found in 33.33% of cases. Hypertension was recorded in 63.89% of cases. The most common presenting complaint was gradual diminution of vision, followed by metamorphopsia and central scotoma. Most cases were pseudophakic (55.55%). Unilateral disease was more common, i.e., 53 (73.61%). Amongst the 19 patients with bilateral disease, 8 (11.11%) had scarred CNVM in one eye. We included only one eye for each patient in our study.

The mean baseline visual acuity was Log MAR.1.061±0.25 (Snellen acuity 5/60). The highest recorded BCVA in the study was 6/12, as depicted in Table 1.

**Table 1:** Distribution of baseline visual acuity *Log MAR - Logarithm of the Minimum Angle of Resolution

Baseline VA	Number of Patients (n)	Decimal VA equivalent	*Log MAR Equivalent
3/60	27	0.05	1.3
4/60	03	0.06	1.2
5/60	05	0.08	1.1
6/60	24	0.1	1.0
6/36	06	0.17	0.8
6/24	03	0.25	0.6
6/18	02	0.33	0.5
6/12	02	0.5	0.3

The central retinal thickness on OCT at baseline ranged between 236 μm - 670 μm with a mean of 349.92±93.78µm. Most of the eys( 37.5%) had a central macular thickness range between 301-350 μm. The baseline characteristics of the types of CNVM in the study group are shown in Table 2.

**Table 2:** Baseline characteristics of the CNVM based on FFA and OCT CNVM: Choroidal neovascular membrane; FFA: Fundus fluorescein angiography; OCT: Optical coherence tomography

Location of CNVM	Number of eyes (%)
Subfoveal	30(41.66)
Juxtafoveal	34(47.22)
Extrafoveal	08(11.11)
Central macular thickness (μm)	
<250	5(6.94)
251-300	27(37.5)
301-350	12(16.67)
351-400	10(13.89)
401-500	13(18.05)
>500	5(6.94)

**Figure 1:**
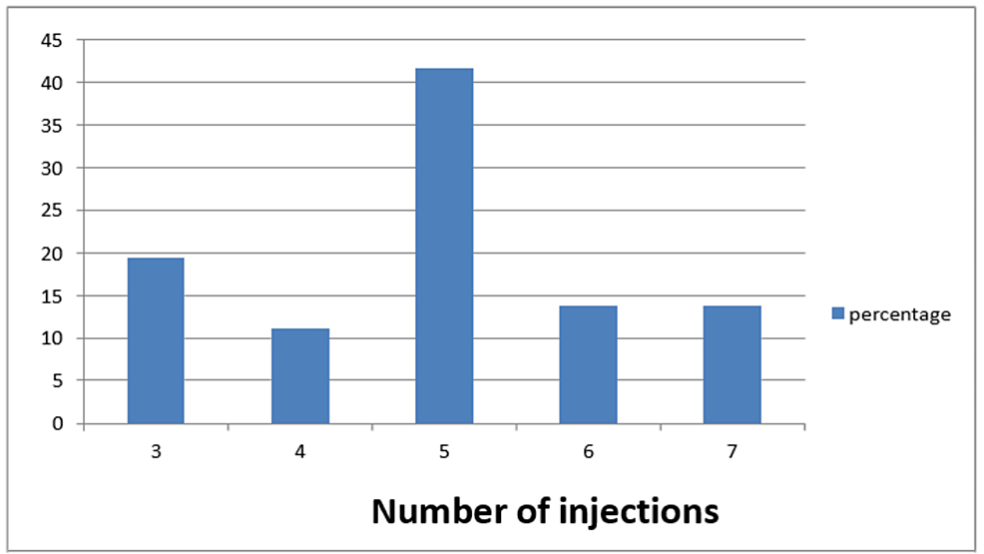
Number of intravitreal Ranibizumab injections given in each eye

The average number of injections given to each eye was five. All the patients received a minimum of three intravitreal Ranibizumab injections at intervals of four weeks in the affected eye, after which the decision to treat further was based on the criteria mentioned before. The total number of injections given was 354. The number of Ranibizumab injections is shown in Fig 1.

Follow-up results

The patients were followed up on the 1st day, 2nd day, 7th day, and 4th week after the procedure, and their BCVA were recorded. Forty-nine patients (68.05%) showed an initial improvement of BCVA of at least one line on Snellen's chart after the first injection. Twenty patients (27.77%) showed no improvement in BCVA over the baseline values after 1st injection.3 patients (4.16%) showed deterioration of one line on Snellen's chart.

Twenty-eight patients (38.8% ) showed improvement in BCVA over a mean follow-up period of nine months (range 6-24 months). Four patients showed moderate visual loss, and BCVA was stable in 34 patients; however, six patients showed no change in BCVA after the completion of injections, as depicted in Table 3.

**Table 3:** Assessment of final visual outcome at the end of the study

Visual acuity	No. of patients(%)	Visual outcome
With> 3 lines gain	28 (38.88)	Improvement
With ≤ 3 lines loss	4 (5.55)	Moderate visual loss
Within 2 lines gain	34 (47.22)	Stable
No change in lines	6 (8.33)	No improvement

The mean visual acuity was log MAR 0.818±0.296 after the third injection. Mean BCVA was improved from 1.061±0.254(5/60) from baseline to 0.787±0.317 on the completion of the study, as shown in Table 4. The mean visual acuity was log MAR 0.787±0.317(6/36).

**Table 4:** Comparison of BCVA (in Log MAR values) after third injection and at the end of the study * statistically significant p value<0.05; Log MAR: Logarithm of the Minimum Angle of Resolution; BCVA: best corrected visual acuity

VA	VA log MAR		p-value from baseline
	Min-Max	Mean±SD	
Baseline	0.2-1.3	1.061±0.254	
After 3rd injection	0.2-1.3	0.818±0.296	<0.001^*^
End of study	0.2-1.7	0.787±0.317	<0.001^*^

The mean central macular thickness at the end of the study was 234.597±45.27µm. Central macular thickness with OCT was at the end of three injections (Ranibizumab) and the end of the study. The mean reduction in CRT was from 349.916±93.78µm at baseline to 234.597±45.27µm. Good response was seen in 37 patients(51.38%), partial response was seen in 29 patients(40.2%), and no response was observed in 6 patients(8.3%), as shown in Table 5.

**Table 5:** Response (reduction in CRT) to Ranibizumab among study participants CRT: Central retinal thickness

Central Macular Thickness	n	Response
Decrease of 100µm in CRT	37	Good response
Decrease of less than 100µm in CRT	29	Partial response
CRT changes by less than 50µm	6	No response

Comparison of CRT between baseline parameters and after the third injection and at the end of the study showed statistically significant differences (depicted in Table 6).

**Table 6:** Comparison of mean CRT at the end of third injection and at the end of the study CRT: Central retinal thickness; OCT: optical coherence tomography **Statistically significant p<0.05

CRT in micron	OCT examination		P value from baseline
	Min-Max	Mean±SD	
Baseline	236-670	349.91±93.78	
After 3rd injection	189-507	270.16±65.91	<0.001^**^
End of study	178-373	234.597±45.27	<0.001^**^

## Discussion

In this study, the prevalence of AMD was strongly age-related-the mean age of patients was70.56±8.56 yrs. The prevalence of early advanced AMD increased with age. According to Beaver Dam Eye Study, AMD in 14.4% of people aged 55-year to 64 years, 19.4% of those aged 65 to 74, and 36.8% of those older than 75, and most of the patients 45(62.5%) were males [13].

Gradual diminution of vision was the most common presentation (90.27%). In their study, Aditya Shudhalker et al. (2015) stated that the most common complaint was decreased vision (94.5%). Smoking has been the one modifiable risk factor consistently linked with an increased risk of age-related macular degeneration [14]. Smoking was present in 24(33.33%) patients.

The Rotterdam study found that the higher the pack-year smoked, the higher the risk of developing neovascular AMD 6.6-fold compared with non-smokers [15]. Smoking was not associated with risk for wet AMD development in this study. This may be due to the female's non-smoking habit in the study's locality. Hypertension (Blood pressure>140/80) was present in 46 patients (63.89%). Hyman et al. identified an association between moderate to severe hypertension and an increased risk of developing neovascular age-related macular degeneration [16]. The Framingham eye study found systolic blood pressure≥170 mmHg [17]. Forty patients (55.5%) were pseudophakic. There were no aphakic patients.

The Blue Mountains eye study and the Beaver Dam eye study found an increased risk of developing AMD in eyes that had undergone cataract surgery [18]. We included only one eye per patient to avoid bias. Classic lesions were predominating (52.78%) classic. Among all the patients, thirty (41.66%) were subfoveal, and Juxtrafoveal in location was predominant thirty-four (47.22%).

Neovascular AMD continues to be a significant cause of visual loss throughout the world. While the advent of anti-VEGF drugs offered new hope to patients with nvAMD, the frequency of injection and requirement for long-term treatment present significant financial and logistical burdens on both patients and the healthcare system. Gene therapy is an ongoing promising alternative to anti-VEGF injections. Gene therapy aims to provide a one-and-done treatment by helping the eye make its own anti-VEGF medicine. Two different methods are under investigation. One injects the gene therapy underneath the retina in a surgical procedure. The other injects it into the eye just like a routine anti-VEGF treatment and is done. Thus, gene therapy may represent the next frontier of treatment for many ocular diseases [19].

The LUMINOUS study with Six thousand two hundred and forty-one neovascular age-related macular degeneration patients treated with Ranibizumab found the incidence of ocular/nonocular adverse events of 8.2%/12.8% and serious adverse events were 0.9%/7.4%, respectively were recruited [20].

One month into the study, a notable enhancement in the mean Best Corrected Visual Acuity (BCVA) was observed, a benefit that was sustained throughout the research. This enhancement in visual acuity correlated with anatomical improvements in the macula, as detected by Optical Coherence Tomography (OCT). Rosenfeld and colleagues observed comparable outcomes in eyes that received ranibizumab treatment [21]. The group's visual acuity showed improvement compared to the baseline measurements, with 100% of ranibizumab-treated patients maintaining or improving or deteriorating vision at the end of the 24 months.

Mean visual acuity (VA) improved from 1.061±0.25 (5/60) to 0.787±0.317(6/36). This was statistically significant (P <0.001). Visual acuity outcome ranged from good (gain of > 3 lines) in 28(38.88%) patients to stable (gain within two lines) in 34(47.22%) patients, four (5.55%) patients showing deterioration, and 6 (8.33%) patients with no change in visual acuity during the period of study. The MARINA and ANCHOR study population of patients prevented the loss of 15 letters (3 lines) at 12 months was 94.5%, 96.4%, and 97.5%, respectively, with 0.3mg of intravitreal Ranibizumab [22].

The primary endpoint of visual acuity, the population of patients gaining> 3 lines, was 38.88%. In the MARINA and ANCHOR & PrONTO study, the population of patients gaining >15 letters at 12 months was 33.8%, 40.3%, and 35 %, respectively. Results of MARINA, ANCHOR, and FOCUS 65 studies have clearly shown that there is always a stage of significant initial gain in vision in the first three months [23].

During the initial treatment phase, 216 injections were given, and 138 injections were given during the remainder of the study. The 138 injections administered during the study were specifically for cases where there was a recurrence of subretinal fluid or cystic changes in the macula within a previously dry lesion. Injections were also given when there was an increase in central retinal thickness (CRT) of more than 100 micrometers or when there was a decrease in visual acuity by five or more letter scores associated with signs of subretinal fluid (SRF) or intraretinal fluid as evidenced by Optical Coherence Tomography (OCT). Additionally, injections were provided in instances of new hemorrhage occurrence. On average, 5.6 injections were administered to each participant throughout the study. At 12 months, patients required a mean of 5.6 injections (compared with 13 in MARINA and ANCHOR) [23]. In the PrONTO study, 17.9% of patients did not require further treatment after the initial three monthly injections [24]. However, in this study, 14 patients (19.44%) did not require further treatment after three injections. This supported the OCT-guided treatment regimen rather than compulsory monthly injections.

There was a prominent decrease in macular thickness as measured by OCT. Most of the change was seen by the 1-month follow-up but continued throughout the study. Mean CRT decreased from 349.91± 93.78μm at baseline to234.59±45.27μm (P < 0.001). This was statistically significant.

Optical Coherence Tomography (OCT) revealed that subretinal fluid (SRF) tended to resolve more quickly than retinal edema, while pigment epithelial detachment (PED) took the longest to resolve. The study did not employ quantitative measures for SRF and PED. Instead, retinal edema was quantitatively assessed by measuring the central macular thickness (CMT). The presence of SRF, PED, or cystoid edema serves as an indicator of disease activity. Although lacking quantitative measurements, the presence of these conditions has been widely utilized as a criterion for retreatment in numerous studies. Our study adhered to this approach, initiating retreatment based on the presence of these signs rather than on quantifiable metrics.

The initial indication of recurrence is typically manifested as subretinal fluid or cystic changes in the macula, often preceding any subjective visual symptoms reported by the patient or observable changes via biomicroscopy. In most instances, a single reinjection of intravitreal Ranibizumab was sufficient to re-establish a dry macular state or reduce the central retinal thickness (CRT) to the lowest level previously recorded.

At the end of the study, all 72 eyes showed improvement in central macular thickness, 37 patients showed a reduction in thickness ≥100μm, 29 patients showed a decrease of central retinal thickness less than 100μm, and six patients showed a change of ≤50μm. All the eyes had marked reduction or absence of leakage from the CNV on angiography.

The ocular side effects of intravitreal Ranibizumab appeared to be quite low. In this study, there were no systemic or local complications reported. None of our cases had any evidence of any raised intra-ocular pressure on the first postoperative day or later; no patient complained of any diminution of vision in the immediate post-op period, and in no patient, detailed indirect ophthalmoscopy revealed any hemorrhage or retinal break. This was in accordance with the FOCUS and PrONTO study, which reported a low incidence of ocular and systemic side effects following intravitreal Ranibizumab [25].

None of the patients developed retinal tears, retinal detachment, glaucoma, uveitis, or endophthalmitis. There were no systemic side effects such as stroke, myocardial infarction, hypertension, proteinuria, and congestive heart failure.

There are certain limitations to our study. It is a single-center hospital-based study conducted during the peak COVID period, due to which the number of patients attending the retina clinic was few. A multicentric study with a greater number of cases can improve the external validity of the study.

## Conclusions

Qualitative OCT is the most sensitive and practical assessment tool to determine anatomic response to treatment. It should be used as an adjunct to clinical examination, decreasing the injection burden without sacrificing improvements in visual acuity. Nevertheless, it is imperative to conduct larger trials with extended follow-up periods to validate these findings definitively.
